# Comparison of arthroscopy versus percutaneous radiofrequency thermal ablation for the management of intra- and juxta-articular elbow osteoid osteoma: case series and a literature review

**DOI:** 10.1186/s12891-022-05244-6

**Published:** 2022-03-25

**Authors:** Igor Knežević, Ivan Bojanić

**Affiliations:** 1 Department of Orthopaedic Surgery, University Hospital Centre Zagreb, Šalata 6-7, 10 000 Zagreb, Croatia; 2 School of Medicine, University of Zagreb, Šalata 2, 10 000 Zagreb, Croatia

**Keywords:** *Elbow*, *Arthroscopy*, *Radiofrequency ablation*, *Intra-articular*, *Tumours*, *Benign neoplasms*, *Osteoid osteoma*

## Abstract

**Background:**

Today, intra-articular and juxta-articular osteoid osteomas are treated with arthroscopy and radiofrequency thermal ablation. However, for the case of an elbow joint, arguments are made for the use of a minimally invasive technique to be the optimal choice. This study aims to analyse our experiences of arthroscopically treated elbow osteoid osteomas and to compare it with the published results of both techniques.

**Methods:**

The retrospective study analyses the patients who underwent elbow arthroscopy ablation of an elbow osteoid osteoma at a single institution from January 2014 until March 2020. Clinical and diagnostic features, success and treatment failure rates, complications and tumour recurrence rates were all compared to 13 studies of intra-articular elbow osteoid osteoma arthroscopic ablation and 15 studies involving radiofrequency thermal ablation of intra-articular osteoid osteoma within different joints.

**Results:**

Four males and two females, with a mean age of 19.3 years, were encompassed. All the patients had immediate postoperative pain relief and improved range of motion. No tumour recurrences were observed during a median of 21.7 months. The literature review yielded 86.4% success rate, 68.2% successful biopsies, one minor complication and no recurrences following the arthroscopic ablation of an elbow osteoid osteoma; while radiofrequency thermal ablation of an intra-articular elbow osteoid osteoma yielded 96.3% success rate, 33.3% successful biopsies, no complications and 3.7% recurrence rate.

**Conclusions:**

Our results are consistent with the published literature proving that arthroscopic ablation is an efficient method with low treatment failure rates and no recurrences in treating intra- and juxta-articular elbow osteoid osteomas. Advantages of arthroscopic ablation stem from the ability to visualise and safely deal with the lesion and the joint’s reactive changes resulting in high biopsy rates, no recurrences and better postoperative elbow’s range of motion. Still, the technique selection should be personalised considering the medical expertise of every institution.

## Introduction

Osteoid osteoma (OO) is a benign osteoblastic bone lesion characterised by the formation of a less than 15 mm wide central nidus surrounded by a halo of sclerosis and cortical thickening. It constitutes 10 to 14% of all benign bone tumours and up to 3% of primary bone tumours [1]. Intra- and juxta-articular OOs are rare compared to extra-articular OOs; they constitute approximately 10 to 20% of all OO cases [2, 3]. The elbow region is involved in about 4% of all OO cases [4, 5]. Furthermore, setting an early accurate diagnosis of intra- and juxta-articular OO poses quite a challenge due to the inconsistent and often misleading symptoms [2, 5–7].

Since 1930, when Bergstrand first described the tumour, and 1935, when Jaffe further defined and categorised it, the OO treatment usually implied an open surgical resection or curettage [1, 7–11]. In 1986, Heuijerjans et al. [12] pioneered the first knee OO arthroscopic treatment. Almost ten years later, Resnick et al. [13] described arthroscopic ablation of OO in the neck of the talus. It did not take long for others to bring the arthroscopic technique for treating OO to different joints. In 2006, the first arthroscopic assisted ablation of an elbow OO was described by Franceschi et al [14]. On the other hand, Rosenthal et al. [15] in 1992 were the first to introduce radiofrequency thermal ablation (RFA) for the treatment of OO [16]. Since then, RFA has proven to be effective and safe for managing extra-articular OOs [3, 16]. However, in the case of intra- and juxta-articular OOs, especially located in close vicinity of neurovascular structures and cartilage, as in the case of the elbow joint, it is discussed which minimally invasive technique is an optimal choice [3, 16–23].

This study aims to analyse preoperative symptoms, treatment effectiveness and postoperative complications for managing intra- and juxta-articular elbow OO with arthroscopic ablation. In addition, we set to compare our results to the ones available in the literature for arthroscopic ablation of intra- and juxta-articular elbow OO as well as to the results following RFA of the intra-articular OO.

## Methods

Following the institutional review board's approval and informed consent, we conducted a retrospective study of all the patients who underwent elbow arthroscopy for the ablation of an elbow OO from January 2014 until March 2020 at a single institution.

Available medical records were scoured for demographic information, including age and gender, as well as clinical characteristics, such as symptoms, time of the onset of the symptoms, history of previous elbow trauma or surgical interventions, intraoperative findings, results of histopathological analysis (HPA) and postoperative complications. Beside plain radiographs, additional radiographic studies, encompassing computed tomography (CT) and magnetic resonance imaging (MRI) reports were also available for analysis. The Mayo Elbow Performance Score (MEPS) was used to estimate the operated elbow function before the arthroscopic procedure and at the final follow-up. The second patient in the series was not evaluated with MEPS because of her age and inability to comprehend the process. The compiled records were reviewed in July 2021 by an independent examiner who was not involved in the patients' care.

**Fig. 1:**
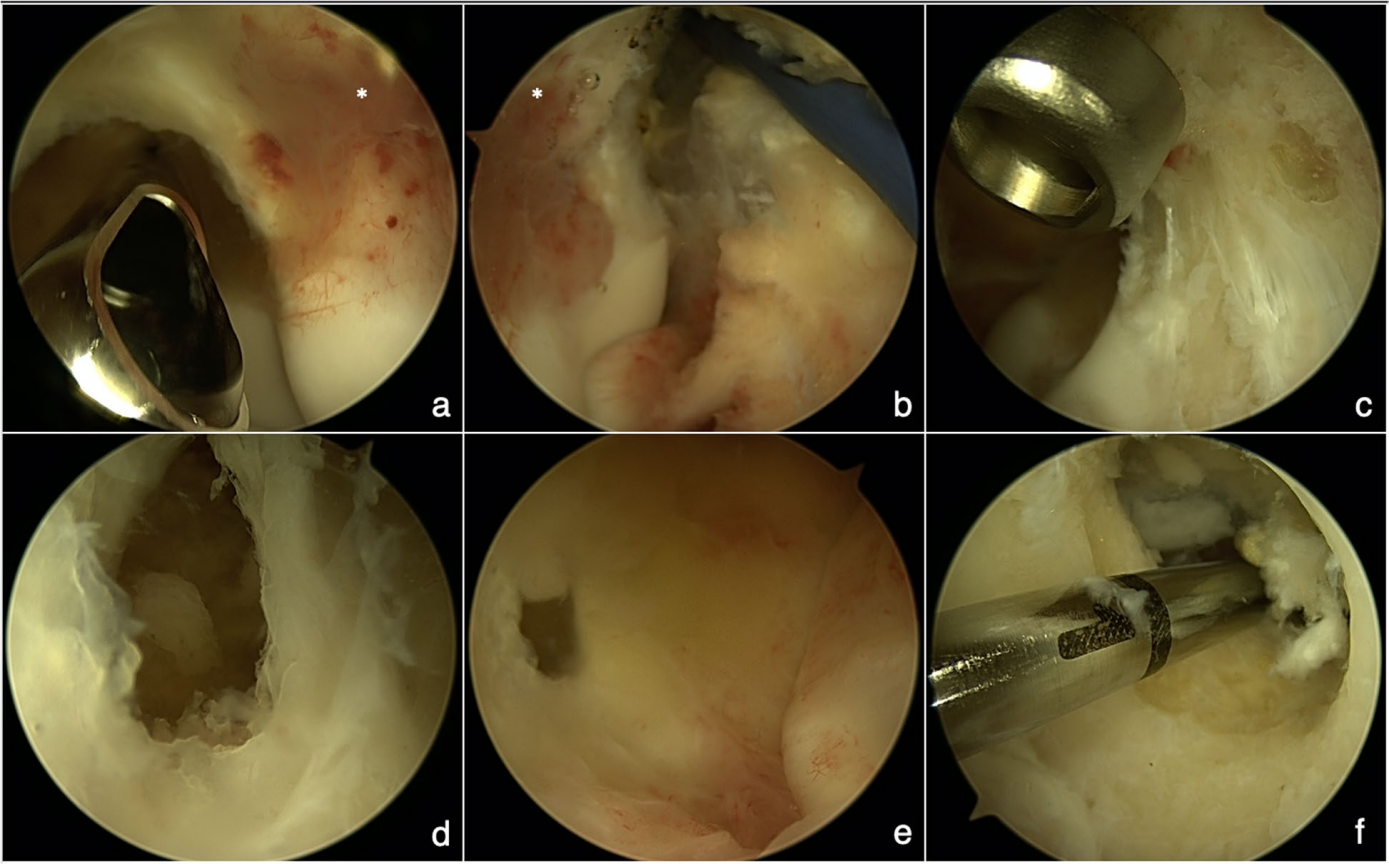
Intra-operative images demonstrating complete arthroscopic ablation of coronoid fossa/olecranon fossa osteoid osteoma enabled by easy visualisation and the use of various arthroscopic tools. **a** an osteoid osteoma (*) at coronoid fossa site surrounded by mild synovitis shown from the anteromedial arthroscopic portal; **b** the osteoid osteoma (*) at coronoid fossa site shown from the anterolateral arthroscopic portal with anterior capsulotomy underway; **c** the coronoid fossa after the biopsy and curettage of the lesion shown from the anteromedial arthroscopic portal; **d** the bone defect after the lesion ablation showing a communication in between coronoid fossa and olecranon fossa visualised from anterior elbow compartment; **e** the bone defect after the lesion ablation showing a communication in between olecranon fossa and coronoid fossa visualised from posterior elbow compartment; **f** the bone defect after the lesion ablation showing the extend of communication in between olecranon fossa and coronoid fossa visualised from posterior elbow compartment

The surgeries were performed consistently by a single surgeon with the patient in a prone position and under general anaesthesia in line with Baker and Jones's technique [24]. Standard 4.0-mm 30° arthroscope was used in all except one case where due to a small child’s elbow, a 2.7-mm 30° arthroscope had to be utilised. All of the patients received perioperative antibiotic prophylaxis in the form of three intravenous Cephazolin doses. An ipsilateral upper arm tourniquet was applied in all cases. Anterior elbow compartment was visualised through proximal anteromedial and proximal anterolateral portals. In cases where the joint's posterior aspect needed to be accessed, direct posterior, posterolateral, and direct lateral portals were used. The lesion is commonly seen as an indigo-coloured region of velvet-textured trabecular bone surrounded by whiter sclerotic bone. A tissue sample was obtained using the curette and arthroscopic grasper tool. Special care was taken to make sure that the specimen was not additionally damaged. Each operation yielded a tissue sample that underwent the latter independent HPA. Motorised arthroscopic tools were then used to remove the remainder of the tumour all up to the healthy bone (Fig. 1). Night-time splinting in elbow extension was postoperatively applied and mandated for the first three postoperative weeks to prevent keeping the elbow in flexion for a prolonged time during nights thus promoting elbow extension. Passive and active-assisted elbow movements were encouraged from the first postoperative day. The follow-up was initially arranged for 3, 6, and 12 weeks after the procedure, followed by a yearly appointment.

We used the same search strategy and inclusion criteria presented by recent 2020 systematic reviews by Ge et al. [20] about arthroscopic management of intra- and juxta-articular osteoid osteoma of the upper extremity and by Lindquester et al. [16] about percutaneous thermal ablation for the treatment of osteoid osteoma to expand the research for additional English language publications in PubMed and Embase up to August 6^th^, 2021 [14, 25–30]. The only RFA studies included were those of authors’ explicitly stating treatment of intra-articular or intra-capsular OO, nevertheless, only two studies, Papagelopoulos et al. [44] and Albisinni et al. [4], consisted exclusively out of cases involving intra-articular OO. Relevant data was then extracted, recorded and analysed by the same investigators using Microsoft Excel 2019 (Microsoft®, Redmond, WA).

Treatment success was defined as the absence of characteristic preoperative pain and improvement on preoperative joint range of motion (ROM). Treatment failure was defined as the persistence of typical preoperative pain or limiting postoperative joint contracture that required an additional procedure with a possible asymptomatic period of over two months [3, 16, 31, 32]. Recurrence of the lesion was considered when specific pain reoccurred in the follow-up period after two or more months without the symptoms [3]. Complications following elbow arthroscopy were categorised as minor or major as published by Nelson et al [33]. Complications that did not require treatment or did not have any consequences after RFA were classified mild, whilst ones that did require intervention were noted as severe [34, 35].

## Results

In six consecutive years, six patients, four males and two females, with a mean age of 19.3 years (range 5 to 33), had been diagnosed with the elbow OO and have undergone arthroscopic ablation. Demographic information and the history of previous elbow trauma, duration, and the character of the preoperative symptoms, intraoperatively confirmed site of the lesion, MEPS trends, and postoperative results are presented in Table 1. Limited elbow ROM and pain relief by nonsteroidal anti-inflammatory drugs (NSAIDs), were predominant preoperative symptoms in 83.3% of cases. The patients were primarily misdiagnosed with monoarticular inflammatory arthritis (80.0% of cases), which caused delay from the first appearance of the symptoms to the surgery in a median of 21.6 months. The first two cases in the series had previous unsuccessful open elbow surgery to alleviate symptoms 2 years and 2 months before the arthroscopic procedure. The first patient had undergone a previous open elbow OO ablation attempt via the posterior approach. However, the pain remained and he developed postoperative elbow contracture. The second case involved a child whose ambiguous symptoms called for an open biopsy of the olecranon fossa region due to a preliminary synovitis diagnosis. Subsequent arthroscopic ablation completely alleviated elbow pain and restored full ROM. Arthroscopic ablations in our cases were not considerably hindered by previous surgeries, nonetheless, it did warrant an anterior capsulotomy to rectify flexion contracture in the first case and partial synovectomy in the second case. In all, except one case, the impartial HPA confirmed the diagnosis of OO (83.3%). Due to inaccessible tumour location at the trochlea site and the use of motorised arthroscopic tools, one sample had been too fragmented for HPA. In that case, the help of intraoperative fluoroscopy was needed to locate the lesion site. The patients were monitored for a median of 21.7 months after the arthroscopic operation. In that period, no tumour recurrence was observed. All the patients had immediate pain resolution. One of the previously operated patients developed postoperative cubital tunnel syndrome, even though elbow pain diminished and full elbow ROM was restored. This could be attributed to ulnar nerve overstretching due to increased postoperative elbow flexion in addition to abundant postoperative scarring contributed by previous open elbow surgery that we failed to anticipate. Therefore, submuscular anterior transposition of the ulnar nerve was successfully performed 16 months after the arthroscopy. Another patient's preoperative elbow flexion contracture has improved but not entirely corrected following the procedure. At the final follow-up appointment, the mean postoperative was MEPS was 95 ± 7.1 compared to preoperative MEPS of 52 ± 16.8.

**Table 1:** Summary of demographic information, clinical presentation, preoperative and postoperative features about the patients involved in this study

Gender and age (years)	History of elbow trauma	Duration of symptoms until the accurate diagnosis (months)	Symptoms	Preoperative MEPS	Preoperative misdiagnosis	Intraoperative fluoroscopy used	Site of the lesion	Histopathological analysis confirmed OO	Postoperative MEPS	Recurrence during the follow-up period (months)	Postoperative elbow status and complications
M (33)	NO	26^a^	persistent pain (relieved by NSAIDs); limited ROM	55	NR	NO	posterior capitellum	YES	85	NO (87)	elbow pain resolution without ROM limitations, ulnar nerve transposition was performed 16 months after arthroscopic surgery
F (5)	YES	12^b^	intensified nocturnal pain (relieved by NSAIDs); limited ROM; joint oedema	NA	monoarticular inflammatory arthritis	NO	olecranon fossa	YES	NA	NO (66)	elbow pain resolution without ROM limitations
M (31)	NO	25	intensified nocturnal pain (relieved by NSAIDs); limited ROM	40	monoarticular inflammatory arthritis	YES	trochlea	NO^c^	90	NO (25)	elbow pain resolution, improved ROM with residual 30° extension contracture
M (18)	YES	18	pain after waking up in the morning; limited ROM	70	posteromedial elbow impingement syndrome	NO	olecranon fossa	YES	100	NO (19)	elbow pain resolution without ROM limitations
F (15)	NO	4	intensified nocturnal pain (relieved by NSAIDs); limited ROM	30	monoarticular inflammatory arthritis	NO	coronoid fossa / olecranon fossa	YES	100	NO (17)	elbow pain resolution without ROM limitations
M (14)	NO	31	persistent pain (relieved by NSAIDs); joint oedema	65	monoarticular inflammatory arthritis	NO	olecranon fossa	YES	100	NO (17)	elbow pain resolution without ROM limitations

*M* Male, *F* Female, *NR* Not Reported, *NA* Not Applicable, *NSAIDs* Nonsteroidal Anti-Inflammatory Drugs, *ROM* Range Of Motion, *MEPS* Mayo Elbow Performance Score, *OO* Osteoid Osteoma

^a^unsuccessful open elbow surgery 2 years before the arthroscopic surgery, ^b^unsuccessful open elbow surgery 2 months before the arthroscopic surgery, ^c^fragmentation of the sample caused by the use of a motorised arthroscopic tool

A review of the literature regarding arthroscopic elbow OO ablation produced 13 studies from 2006 to 2021 (Table 2) [14, 23, 25–27, 36–43]. The studies involved a total of 23 patients, predominantly male (5 to 1). The mean age was 27.2 ± 9.0 (range, 15 to 48) years. The most prominent preoperative symptom was limited elbow ROM, presented in 91.3% of cases, followed by pain relief on NSAID use, and nocturnal pain in 82.6% and 65.2% of cases, respectively. The delay from the first symptoms to the surgery has a range from 6 to 120 months, with a median of 21.7 months. The lesion was mainly located inside the olecranon fossa in 8 (34.8%) cases. Biopsy was performed in 22 cases, while OO was histologically confirmed from 68.2% of samples. Intraoperative fluoroscopy was used in 13 cases. During the median follow-up period of 24.0 (range, 1.5 to 78) months, 3 (13.0%) treatment failures resulted in residual pain due to incomplete resection or inadequate preoperative elbow contracture correction, prompting an open surgery. No recurrences were noted. Major elbow arthroscopy complications were not recorded. One minor complication (4.3%) developed as the onset of mechanical elbow pain in a patient with long-standing elbow contracture and synovitis which did not require additional surgery.

**Table 2:** Summary of the available literature about the use of elbow arthroscopy for the treatment of intra- and juxta-articular osteoid osteoma, modified and updated from Ge et al. [20]

Study (year)	Sample size	Gender and age (years)	History of elbow trauma	Duration of symptoms until the accurate diagnosis (months)	Symptoms	Pre-operative misdiagnosis	Fluoroscopy used	Site of the lesion	Histopathological analysis confirmed OO	Recurrence during the follow-up period (months)	Post-operative elbow status and complications
**Franceschi et al** **(2006) **[14]	1	M (42)	YES	120	intensified nocturnal pain (relieved by NSAIDs); limited ROM; joint oedema	post-traumatic periostitis	YES	olecranon fossa	YES	NO (46)	the patient returned to full activity and full-time employment
**Trebse et al** **(2007) **[36]	1	M (42)	NO	18	persistent pain; limited ROM; joint oedema ^c^	osteochondroma	NO	radial head	YES	NO (24)	elbow pain resolution, with improved residual 30° pronation contracture
**Nourissat et al** **(2007) **[37]	2	M (20)	NR	NR	no pain; limited ROM	NR	NO	posterior capitellum	YES	NO (8)	elbow pain resolution without ROM limitations
		M (27)	NO	36	intensified nocturnal pain (relieved by NSAIDs); limited ROM	epicondylitis	NO	trochlea	YES	NO (1.5)	residual pain prompted an open surgery due to incomplete resection of the tumour two weeks after surgery
**Zupanc et al** **(2007) **[38]	1	M (42)	YES	30	intensified nocturnal pain (partially relieved by NSAIDs); limited ROM ^c^	epicondylitis	YES	posterior capitellum	NO^a^	NO (12)	elbow pain resolution without ROM limitations
**Font Segura et al** **(2013) **[39]	1	F (15)	YES	24	persistent pain (partially relieved by NSAIDs); limited ROM ^¤^	NR	NO	olecranon fossa	YES	NO (24)	elbow pain resolution without ROM limitations
**Glanzmann et al** **(2013) **[43]	1	M (20)	NO	42	NR	NR	NR	coronoid fossa	YES	NO (36)	NR
**Akpinar et Circi** **(2017) **[40]	1	F (23)	NO	6	persistent pain (partially relieved by NSAIDs)	synovitis	NO	coronoid fossa	YES	NO (36)	elbow pain resolution returned to full activity
**Kamrani et al** **(2017) **[23]	10	M (20)	NR	22	nocturnal pain (relieved by NSAIDs); limited ROM^b^	NR	YES	olecranon fossa	YES	NO (78)	
		M (19)	NR	36		NR		olecranon fossa	NO^a^	NO (54)	elbow pain resolution, with residual limited ROM, which prompted an open surgery
		M (28)	NR	18		NR		trochlea	NO^a^	NO (72)	altered mechanical elbow pain remained that had not resulted in revision surgery
		M (48)	NR	18		NR		coronoid	YES	NO (68)	
		M (24)	NR	36		NR		coronoid	NO^a^	NO (42)	
		M (28)	NR	36		NR		radial head	NO^a^	NO (30)	residual pain prompted an open surgery due to incomplete resection of the tumour
		M (35)	NR	12		NR		coronoid	NO^a^	NO (20)	
		M (25)	NR	24		NR		coronoid	YES	NO (18)	
		M (24)	NR	16		NR		olecranon fossa	YES	NO (16)	
		M (18)	NR	16		NR		radial head	NO^a^	NO (18)	
**Goyal et al** **(2018) **[41]	1	M (25)	NO	24	persistent pain getting worse after periods of rest and after waking up in the morning (partially relieved by NSAIDs); limited ROM	monoarticular inflammatory arthritis	NO	coronoid fossa	YES	NO (12)	elbow pain resolution without ROM limitations
**Hatta et al** **(2019) **[42]	1	F (17)	NR	12	intensified nocturnal pain; limited ROM	monoarticular inflammatory arthritis	NO	coronoid fossa / olecranon fossa	YES	NO (12)	elbow pain resolution without ROM limitations
**Yano et al** **(2020) **[25]	1	M (26)	NO	11	persistent pain (partially relieved by NSAIDs); limited ROM; joint oedema	monoarticular inflammatory arthritis	YES	olecranon fossa	YES	NO (24)	elbow pain resolution without ROM limitations
**Sridharan et al** **(2021) **[26]	1	M (30)	NO	18	persistent pain (partially relieved by NSAIDs); limited ROM; joint oedema	NR	NR	trochlea	YES	NO (6)	elbow pain resolution without ROM limitations
**Alrassasi et al** **(2021) **[27]	1	F (28)	NR	NR	nocturnal pain (relieved by NSAIDs); limited ROM	NR	NR	olecranon fossa	NR	NO (12)	elbow pain resolution, improved ROM with residual 30° flexion contracture

*M* Male, *F* Female, *NR* Not Reported, *NSAIDs* Nonsteroidal Anti-Inflammatory Drugs, *ROM* Range Of Motion, *OO* Osteoid Osteoma

^a^fragmentation of the sample due to inability to properly visualise the tumour and the use of motorised arthroscopic tools for ablation, ^b^it is reported that all 10 patients in the study had presented with classic symptoms of limited ROM and nocturnal pain that subsides on use of NSAID, ^c^unsuccessful previous open elbow surgery, ^¤^ previous elbow trauma prompted an open surgery with residually limited ROM resulting in ever-increasing elbow contracture

**Fig. 2:**
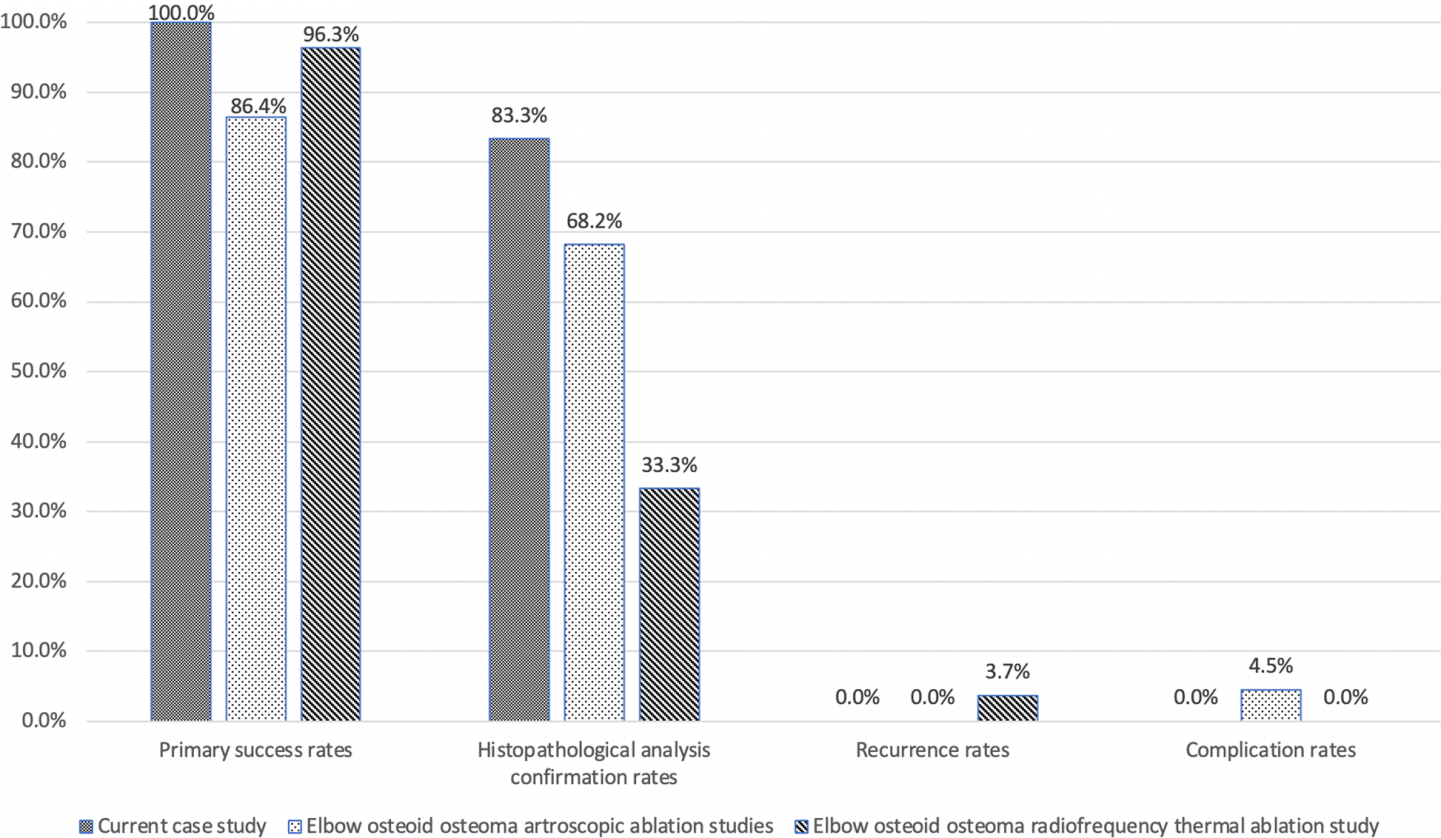
A diagram showing primary success rates, histopathological analysis confirmation rates for osteoid osteoma following attempted biopsies, recurrence and complications rates for six cases in our current study compared to 13 studies exploring arthroscopic ablation of elbow osteoid osteoma as well as Albisinni et al. [4] study involving radiofrequency thermal ablation of elbow osteoid osteoma

A total of 1286 cases involving RFA treatment for OO were analysed, including 198 (15.4%) cases of intra-articular or intra-capsular OO located within various joints counting the elbow joint (Table 3) [4, 19, 28–32, 44–51]. The average age of patients was 19.0 ± 6 (range, 12 to 30) years. Patients mainly presented (73.3%) with increasing nocturnal pain partially or entirely relieved by NSAID. The delay period before the procedure was 19.6 ± 13 (range, 6 to 43) months. When biopsy have been attempted, in 7 out of 15 studies, HPA successfully diagnosed OO in an average of 42.6% of cases. The average primary success rate of the RFA procedure was 94.5%, with a total recurrence rate of 4.1%. In 2.3% of cases, complications were recorded during the average follow-up period of 36.5 ± 23 (range, 12 to 93) months. Severe complications requiring additional intervention was noted in 1.3% of cases, whilst mild complications were present in 1.0% of cases. Comparison of the results for both arthroscopic ablation and intra-articular elbow OO RFA is presented in Fig. 2.

**Table 3:** Summary of the available literature involving use of radiofrequency thermal ablation for the treatment of extra- and intra-articular or intra-capsular osteoid osteoma, modified and updated from Lindquester et al. [16]

Study (year)	Sample size (intra-articular OO)	Average age of the patients (years)	Average duration of symptoms until the accurate diagnosis (months)	Symptoms	Histopathological analysis performed (number of confirmations / number of biopsies)	Average follow-up period (months)	Primary success rate ^a^	Recurrence rate (number of recurrences/ sample size)	Complications
**Ghanem et al** **(2003) **[31]	23 (2)	12	34	intensified nocturnal pain, joint stiffness, limp	YES (1/12)	41	91.3% (21/23)	8.7% (2/23)	2 S—developed asymmetry of joint range of motion^b^ 1 m—transient muscle atrophy
**Papagelopoulos et al** **(2006) **[44]	16 (16)	27	NR	intensified nocturnal pain (relieved by NSAIDs)	NO	30	100% (16/16)	0% (0/16)	5 m—transient pain 1 m—transient paraesthesia
**Peyser et al** **(2007) **[32]	51 (7)	20	11	intensified nocturnal pain (relieved by NSAIDs)	YES (15/32)	24	98.0% (50/51)	2.0% (1/51)	1 S—wound infection
**Peyser et al** **(2009) **[45]	22 (5)	13	12	pain	YES (8/12)	39	95.5% (21/22)	4.5% (1/22)	1 S—subtalar joint degenerative changes 1 m—superficial infection
**Akhlaghpoor et al** **(2010) **[46]	21 (6)	19	43	persistent pain partially relieved by NSAIDs	NO	28	100% (21/21)	0% (0/21)	1 m—skin burn
**Mylona et al** **(2010) **[19]	23 (7)	28	19	intensified nocturnal pain	NO	12	91.3% (21/23)	0% (0/23)	NO
**Al-Omari et al** **(2012) **[47]	30 (2)	15	NR	intensified nocturnal pain (relieved by NSAIDs)	NR	30	93.3% (28/30)	3.3% (1/30)	2 m—skin burn
**Rimondi et al** **(2012) **[48]	557 (65)	21	~ 6 ^c^	persistent pain partially relieved by NSAIDs	YES (95/557)	42	95.7% (533/557)	4.3% (24/557)	2 S—maximum procedure temperature was not achieved 1 m—thrombophlebitis 1 m—skin burn 1 m—broken electrode
**Albisinni et al** **(2014) **[4]	27 (27)	30	30	intensified nocturnal pain (relieved by NSAIDs)	YES (9/27)	67	96.3% (26/27)	3.7% (1/27)	NO
**Cheng et al** **(2014) **[49]	66 (14)	19	NR	NR	NR	53	92.1% (58/63)^d^	7.6% (5/63)^d^	1 S—wound infection
**Garge et al** **(2017) **[50]	30 (4)	13	NR	intensified nocturnal pain (relieved by NSAIDs)	YES (10/18)	NR	96.7% (29/30)	0% (0/30)	1 m—transient interosseous nerve damage following OO ablation of the radial head
**Hage et al** **(2018) **[51]	92 (3)	18	NR	intensified nocturnal pain (relieved by NSAIDs)	NR	93	91.3% (84/92)	6.5% (6/92)	1 S – surgically treated abscess 1 S—pulmonary oedema
**Esteban Cuesta et al** **(2018) **[28]	207 (13)	22	NR	characteristic pain	NR	12	98.1% (203/207)	1.9% (4/207)	3 S—technique failures
**May et al** **(2019) **[29]	43 (26)	12	~ 6 ^e^	intensified nocturnal pain (relieved by NSAIDs), limp	YES (43/84)	12	92.7% (38/41)	NR	1 S – deep tissue infection 1 S – pathological fracture 1 m – transient paraesthesia
**Baal et al** **(2019) **[30]	71 (1)	16	NR	intensified nocturnal pain (relieved by NSAIDs)	NR	27	85.9% (61/71)	14.1% (10/71)	NO

*OO* Osteoid Osteoma, *S* Severe complication, *m* mild complication, *NR* Not Reported, *NSAIDs* Nonsteroidal Anti-Inflammatory Drugs

^a^lack of clinical symptoms and / or radiological presence of the lesion after the RFA procedure, ^b^both cases pretend to intra-articular OO, only locations of the intra-articular OOs are presented, ^c^authors presented that in 55% of cases the period from the first symptoms until the procedure was less than 6 months, ^d^three patients were lost to follow-up, ^in intra-articular cases of OO, an additional triamcinolone acetonide injection was administered, ^e^authors presented that in 57% of cases the period from the first symptoms until the procedure was less than 6 months

## Discussion

Our results show the effectiveness of the arthroscopic ablation for treating intra- and juxta-articular elbow OOs with low rates of treatment failure and no recurrences. Furthermore, with no major complications, it is proven safe for use in the vicinity of joint cartilage and neurovascular structures.

Generally accepted advantages of an arthroscopic procedure in conjuncture with a clear tumour visualisation and sample gathering, as well as, an opportunity to deal with an elbow contracture, it makes this technique a useful option for treatment of intra- and juxta-articular elbow OOs [20, 21, 52, 53]. Cases involving arthroscopic treatment of intra- and juxta-articular elbow OO are scarce. Similarly, the use of the arthroscopic technique for ablation of intra-articular OO in various other joints have proven to be safe and effective. For example, Marwan et al. [53] in a 2015 systematic review involving 10 cases with intra-articular hip OO, showed a 100% success rate, no recurrences and one minor complication following arthroscopic ablation. Ge et al. [21] in 2018, firstly reviewed arthroscopic management of the 27 intra-articular ankle OOs and reported a success rate of 96% without complications and with one recurrence, one year after the procedure. Moreover, in 2020 Ge et al. [20] in another systematic review, examined arthroscopic management of intra‐ and juxta‐articular osteoid osteoma of the upper extremities and concluded that arthroscopic ablation of the shoulder and wrist OOs was successful in 100% of cases with no complications or recurrences. We expanded on their research regarding ablation of intra-articular elbow OO complementing it with three additional studies adding up to a total success rate of 86.4%, no recurrences and one minor complication (4.5%) after arthroscopic ablation of intra- and juxta-articular elbow OOs [14, 23, 25–27, 36–43]. Treatment failures (15.6%) included three patients requiring additional surgical intervention, 2 due to residual pain and one case of residual elbow contracture [23, 37]. Reactive synovitis and adhesions causing joint contracture frequently accompany intra-articular OOs [27, 44, 54, 55]. Elbow arthroscopy enables concurrent biopsy and ablation of the lesion while addressing other conditions like synovitis and joint contracture by performing synovectomy or capsulotomy. [26, 43] This is reflected in increased postoperative performance scores and improved ROM. Our case study's preoperative MEPS of 52 ± 17.0 and postoperative MEPS of 95 ± 7.1 could be compared to Albisinni et al. [4] RFA ablation of the intra-articular elbow OO with preprocedural MEPS of 54.8 ± 14.8 and postprocedural MEPS of 94.6 ± 10.5. Furthermore, they reported that postprocedural full elbow ROM was achieved in 55.5% of cases compared to 81.8% after arthroscopic ablation in our study [4].

On the other hand, RFA has been established as an effective minimally invasive technique for OO treatment even in difficult to reach anatomical regions while providing short procedure duration and hospital stay [22]. Lindquester et al. [16] in a 2020 meta-analysis, reported an overall success rate of 91.9% following RFA of OOs. Both Lanza et al. [35] and Tordjman et al. [3] reported similar results on the treatment failure rate of 5.2% and 8.3%. Recurrence rates vary from 4.1% in our analysis to 5.6% in the available literature [16, 35]. Overall, reported RFA complication rates range from 2.1% to 3.0% [3, 16, 35]. Efthymiadis et al. [55] in a 2021 systematic review and a proportional meta-analysis regarding optimal technique for treating hip intra-articular osteoid osteoma, showed that RFA and percutaneous drilling was associated with two bone fractures, while the arthroscopic approach had no complications. Using CT guided RFA and a matter of radiation exposure in the paediatric population is also something to be aware of [56]. One of the RFA's limitations involve a higher risk of unintentional damage to neural structures or articular cartilage, especially in an environment like joints or spine. Therefore, it is proposed that special precautions should be taken in these cases, or an alternative solution should be pursued [3, 16, 18, 20, 57]. This also tends to be cases where the diagnosis is inconclusive, the nidus of the lesion is closer than 1.5 cm to a neural structure, cartilage or growth plates and cases where previous minimally invasive procedures were unsuccessful [32, 44, 45, 58].

**Fig. 3:**
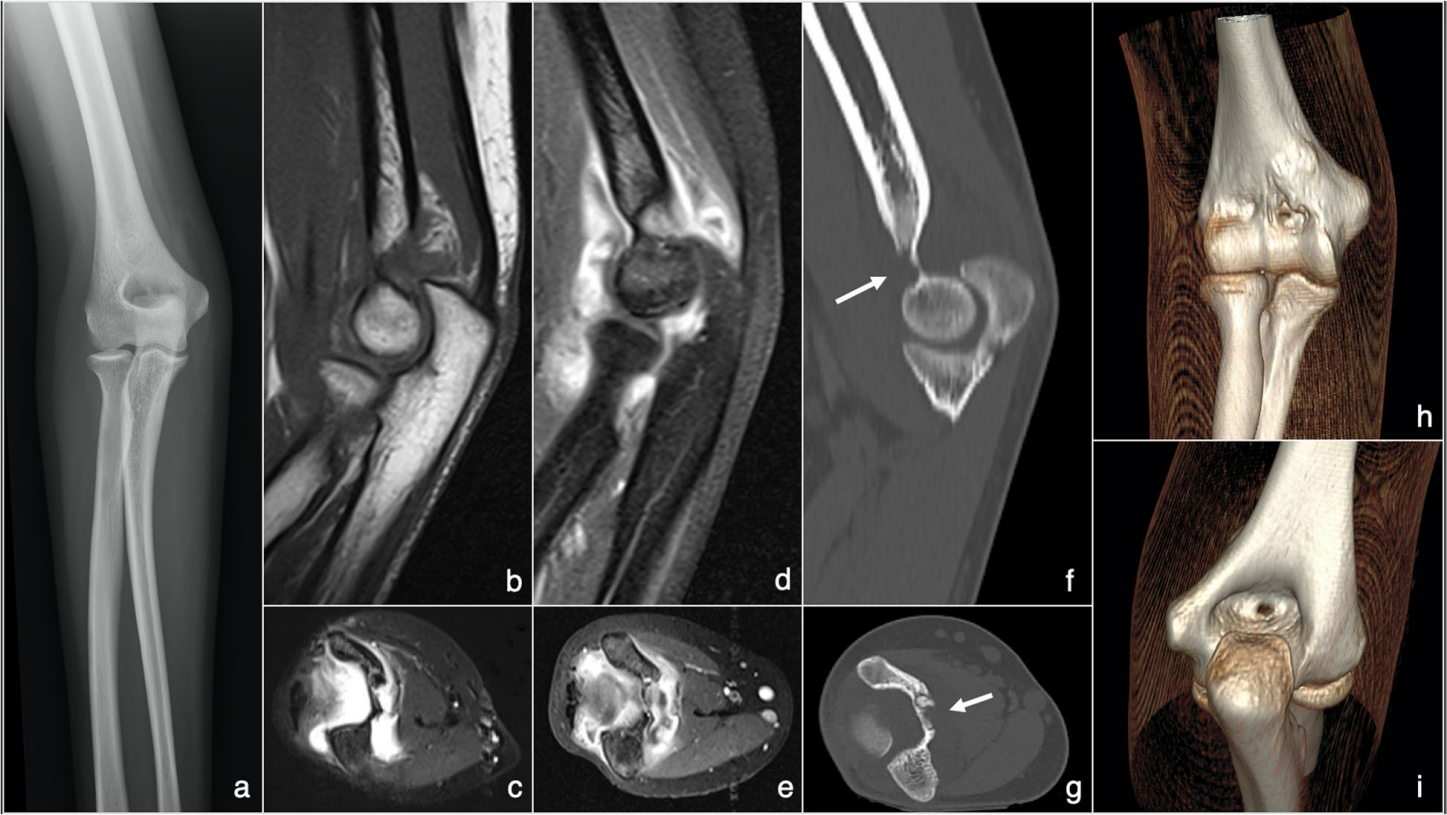
Various radiographic modalities performed on a patient with elbow osteoid osteoma presented in this study: **a** plain radiography anteroposterior projection image of the right elbow affected by osteoid osteoma; **b** sagittal MRI projection image of the right elbow showing mild signs of elbow oedema; **c** axial MRI projection image of the right elbow showing signs of elbow oedema; **d** sagittal magnetic resonance arthrography projection image of the right elbow showing signs of elbow oedema; **e** axial magnetic resonance arthrography projection image of the right elbow showing signs of elbow edema; **f** sagittal CT projection image of the right elbow with an arrow pointing to the osteoid osteoma site in between coronoid and olecranon fossa; **g** axial CT projection image of the right elbow with an arrow pointing to the osteoid osteoma site in between coronoid and olecranon fossa; **h** 3D reconstruction CT image of the right elbow demonstrating osteoid osteoma site from the anterior view; **i** 3D reconstruction CT image of the right elbow showing osteoid osteoma site from the posterior view

Comparing our findings with the published literature, it is evident that intra-articular placement of an elbow OO could lead to non-specific symptoms and prolonged time until the accurate diagnosis. Combining CT scan and high suspicion should lead to an early OO diagnosis (Fig. 3) [2, 5–7, 44, 59–65]. Therefore, histological diagnosis confirmation of the OO appears even more important [2, 16, 48, 66]. Arthroscopy allows reliable lesion sampling before the ablation. In one of our cases, HPA was inconclusive due to the fragmentation of the sample. Zupanc et al. [38] and Kamrani et al. [23] also reported inconclusive HPA due to sample fragmentation resulting in a total of 68.2% HPA confirmations after arthroscopic ablation of elbow OO. However, elbow arthroscopy requires mastery of advanced arthroscopic skills to locate and visualise the tumour. Sometimes, there is a need for specialised equipment like 70° angled arthroscope or an intraoperative use of fluoroscopic assessment [23, 67]. In comparison, using RFA, it is challenging to obtain a proper tissue sample for HPA, resulting in 59.3% OO diagnosis, going down to an average of 33.3% in cases involving intra-articular elbow OOs as reported by Albisinni et al [4, 16, 35]. Thus, there are doubts about the usefulness of biopsy attempst during RFA due to low lesion confirmations rates [16, 31, 32, 46, 68].

Recurrent OO is very unlikely. Therefore, some studies insinuate that it results from an incomplete ablation of the tumour, while others suggest that further investigations should involve topics of long-term regrowth after the procedures or errors in the differential diagnosis [35]. Lanza et al. [35] suggested that biopsy predicts low recurrence rates, but it should not be performed routinely. No recurrences after arthroscopic ablation of elbow OO in addition to higher HPA confirmation rates, might suggest more attention should be focused on obtaining a proper lesion sample. It is evident that complications and arthroscopic procedure failures are apparent immediately or shortly after the initial procedure. Studies about RFA treatment of OOs report that the majority of recurrences occur as pain within the first seven months after the procedure [16, 46, 58].

Some studies relied on postoperative CT or MRI to confirm the complete ablation of the tumour and keeping track of healing patterns [25, 69]. We found that only Yano et at. [25] performed postoperative CT, confirming an appropriate resection of the nidus following an arthroscopic ablation. Nonetheless, we did not see any merit in performing a postoperative CT or MRI scan because all of our patients had their preoperative symptoms resolved immediately after surgery. Furthermore, Lanza et al. [35] suggested that postoperative CT or MRI should be performed more as a precaution and that physicians only loosely carried out post-RFA routine follow-up.

There are some other limitations of this report apart its retrospective nature. Due to limited availability of more valued study designs, the published literature about arthroscopic ablation of an elbow OO includes either case series or case reports, which contribute only level 4 and 5 evidence. This suggests that only uncommon and novel cases have been publicised, which may not give an appropriate image of the elbow OO entity. In addition, most RFA studies involve heterogeneous data combinations of extra-articular and intra-articular OOs within different joints and the use of diverse RFA technique modalities. Therefore, there is a need for further multicentre prospective research comparing the results of both treatments exclusively for intra- and juxta-articular OO. We mainly presented short-term results of the arthroscopic ablation. However, long-term study of the arthroscopic ablation effect on the elbow joint stability or the joint cartilage might also be a topic for future investigation.

## Conclusions

Due to inconsistent symptoms, in combination with ambiguous radiological reports, timely intra- and juxta-articular OO diagnosis remains a challenge. Arthroscopic ablation and RFA have taken a lead as preferred techniques in treating intra- and juxta-articular OOs. However, the advantages of arthroscopic elbow OO ablation are very clear due to the ability to directly visualise and safely deal with the lesion and the joint’s reactive changes, resulting in higher biopsy rates, no recurrences and better postoperative ROM. Our results are coherent with the published literature proving that arthroscopic ablation is an efficient method with low treatment failure rates and no recurrences in treating intra- and juxta-articular elbow OOs. Still, technique selection should be personalised, taking into account the medical expertise of every institution.

## Data Availability

The datasets generated and/or analysed during the current study are not publicly available due to limitations of ethical approval involving the patient data and anonymity but are available from the corresponding author on reasonable request.

## Abbreviations

OO: Osteoid osteoma
RFA: Radiofrequency thermal ablation
HPA: Histopathological analysis
CT: Computed tomography
MRI: Magnetic resonance imaging
MEPS: Mayo Elbow Performance Score
ROM: Range of motion
NSAIDs: Nonsteroidal anti-inflammatory drugs
